# Trichome-Specific Analysis and Weighted Gene Co-Expression Correlation Network Analysis (WGCNA) Reveal Potential Regulation Mechanism of Artemisinin Biosynthesis in *Artemisia annua*

**DOI:** 10.3390/ijms24108473

**Published:** 2023-05-09

**Authors:** Dawei Huang, Guixian Zhong, Shiyang Zhang, Kerui Jiang, Chen Wang, Jian Wu, Bo Wang

**Affiliations:** 1 Guangdong Key Laboratory of Plant Molecular Breeding, College of Agriculture, South China Agricultural University, Guangzhou 510642, China; dawei_huang@163.com (D.H.); zhongguixian2021@163.com (G.Z.); zhangshiyang1997@126.com (S.Z.); jiangkr127@163.com (K.J.); kyle99423@163.com (C.W.); 2 Guangdong Laboratory for Lingnan Modern Agriculture, Guangzhou 510642, China

**Keywords:** trichome, weighted gene co-expression network analysis, hub genes, terpenoid metabolism, *Artemisia annua*

## Abstract

Trichomes are attractive cells for terpenoid biosynthesis and accumulation in *Artemisia annua*. However, the molecular process underlying the trichome of *A. annua* is not yet fully elucidated. In this study, an analysis of multi-tissue transcriptome data was performed to examine trichome-specific expression patterns. A total of 6646 genes were screened and highly expressed in trichomes, including artemisinin biosynthetic genes such as *amorpha-4,11-diene synthase* (*ADS*) and *cytochrome P450 monooxygenase* (*CYP71AV1*). Mapman and Kyoto Encyclopedia of Genes and Genomes (KEGG) pathway enrichment analysis showed that trichome-specific genes were mainly enriched in lipid metabolism and terpenoid metabolism. These trichome-specific genes were analyzed by a weighted gene co-expression network analysis (WGCNA), and the blue module linked to terpenoid backbone biosynthesis was determined. Hub genes correlated with the artemisinin biosynthetic genes were selected based on TOM value. *ORA*, *Benzoate carboxyl methyltransferase* (*BAMT*), *Lysine histidine transporter-like 8* (*AATL1*), *Ubiquitin-like protease 1* (*Ulp1*) and *TUBBY* were revealed as key hub genes induced by methyl jasmonate (MeJA) for regulating artemisinin biosynthesis. In summary, the identified trichome-specific genes, modules, pathways and hub genes provide clues and shed light on the potential regulatory mechanisms of artemisinin biosynthesis in trichomes in *A. annua*.

## 1. Introduction

*Artemisia annua* L. (Sweet Wormwood) is a medicinal herb that is famous for artemisinin, a sesquiterpene with anti-malarial activity. The biosynthetic pathway of artemisinin begins with amorpha-4,11-diene, which is generated from farnesyl diphosphate (FPP) with the help of glandular trichome-specific enzyme amorpha-4,11-diene synthase (ADS) [1]. Subsequently, another trichome-specific enzyme, a multi-function cytochrome P450 monooxygenase (CYP71AV1) catalyzes amorpha-4,11-diene to artemisinic alcohol, artemisinic aldehyde and artemisinic acid by three stepped reactions [2]. Artemisinic aldehyde Δ11 (13) reductase (DBR2) catalyzes the transform of artemisinic aldehyde to dihydro artemisinic aldehyde, and aldehyde dehydrogenase (ALDH1) converts (dihydro) artemisinic aldehyde into (dihydro) artemisinic acid [3]. Although researchers have found that artemisinin biosynthesis occurs in trichomes [4], the molecular basis of trichomes is not fully elucidated yet in *A. annua*.

The trichome is the extension of the epidermis and consists of a single cell or a group of cells that vary in size, morphology, structure and function. It can be stellate, squamous or glandular, and it presents on most surfaces of vascular plants to protect plants from viruses, herbivores, ultraviolet light and so on [5]. In addition to defending against herbivores and resisting various stresses, trichomes have a wide range of metabolic processes and produce, store and release a variety of substances that are not necessarily essential for the trichomes’ normal development, including isoprenoids, phenylpropanoids, alkaloids and O-acyl sugars [6]. Many isoprenoids are believed to be involved in defensive mechanisms against herbivores and arthropods, as well as being crucial molecules for maintaining human health. Trichome-specific transcription factors, such as AaGSW1, AaORA and AaTCP15, can form a module to influence artemisinin biosynthesis in *A. annua* [7]. In addition, trichome initiation and development also influence artemisinin accumulation. R2R3-MYB and HD-ZIP IV are the majority transcription factors involved in glandular trichome initiation in *A. annua* [8]. A R2R3-MYB transcription factor, *AaMIXTA1*, which is expressed predominantly in the glandular trichome, could form a regulatory complex with *AaHD8* to regulate glandular trichome initiation in *A. annua* [9,10]. HOMEODOMAIN PROTEIN 1 (*AaHD1*) could control both glandular and nonglandular trichome initiations by promoting the expression of *AaGSW2* [11]. Overexpression of these trichome initiation genes, such as *AaSEPALLATA1*, *AaWIN1* and *AaSPL9*, could improve the artemisinin content in *A. annua* [12,13,14]. Thus, the investigation of trichome-specific transcripts may be a successful strategy for the selection of candidate genes to improve useful trichome-specific secondary chemicals in the future.

To elucidate the metabolic capacities of *A. annua* trichomes, researchers have made a great effort in the trichome transcriptome in *A. annua*. Wang et al. built up the first glandular trichome expressed sequence tag datasets (ESTs) by extracting the glandular trichome from the surface of flower buds [15]. In addition, photosynthesis-related genes and ABC transporters have been found enriched in *A. annua* glandular trichomes by comparison with trichome ESTs in the public TrichOME database [15]. Using EST technology as well, Graham et al. collected the glandular trichome from the *Artemisia* hybrid, obtained the ESTs using the Roche 454 platform, and selected key genes associated with artemisinin metabolism and trichome density by quantitative trait loci (QTL) mapping [16]. Furthermore, glandular and filamentous trichomes were separated by laser pressure catapulting, and sequenced using Illumina RNA-Seq [17]. It was shown that lipid, terpenoid backbone (MVA and MEP) and most terpenoid biosynthesis pathways were significantly upregulated in glandular trichomes, while specific terpene metabolic pathways exist in the filamentous trichomes as well [17]. With the development of DNA sequencing technology, the *A. annua* genome has been published, and mixed trichome cells were collected as one of the reference transcripts [18]. This reference genome improved the gene assembly and annotation of *A. annua*, providing a variety of information for the artemisinin-related investigation. Therefore, a trichome-specific analysis will further extend more valuable information for artemisinin biosynthesis.

In this study, the SPM algorithm was used to perform the trichome-specific analysis in *A. annua* using RNAseq data, and 6646 trichome-specific genes were identified. Further results showed that pathways related to energy, terpenoids and fatty acid were significantly enriched in the trichome-specific gene set. Using WGCNA analysis, gene networks related to terpenoid biosynthesis were identified as blue module, and some hub factors possibly related to artemisinin biosynthesis were found. These candidate genes are involved in the transfer of carboxyl and methyl, the transportation of amino acids, and SUMO reactions, which are regulated by methyl jasmonate (MeJA) treatment. Our transcriptome analysis highlights the trichome-specific metabolic activities in *A. annua*, especially the genes involved in the artemisinin biosynthesis, providing putative gene candidates for further investigation.

## 2. Results

### 2.1. Assembly and Annotation of the Transcriptome Data

To obtain a further view of transcription relevant to *A. annua* trichomes and other tissues (flowers, young leaves, stems, old petioles, etc.), 12 samples were sequenced using the Illumina platform. A total of 96.15 Gb clean data was obtained, with at least 6.40 Gb clean data for each sample (more than 93.34% of bases scored over Q30). Then, the clean data was mapped to the reference genome, with the mapping ratio ranging from 86.22% to 89.50%. According to the mapped results, 12,871 novel genes were identified, 8364 of which were function annotated. The alignment results were used for gene expression analysis.

In addition, seven tissues’ transcriptomes (trichome, flower, bud, young leaf, stem, root and seed) were obtained from the NCBI database for trichome-specific expression analysis. Then, reads in seven tissues’ transcriptomes were filtered and trimmed using Trimmomatic. We used genes in our sequencing data as a reference index to calculate the TPM of each gene by the quasi-mapping-based mode of Salmon. In total, the expressing state of 79,576 genes (99.82% of all genes in the reference gene set) was collected. All genes were annotated via BLAST using the NR database, Swiss-Prot database, GO database and KEGG database. There were 89.12%, 65.48%, 61.98%, 26.86%, 81.02% and 49.11% of sequences paired with genes in the Nr database, Pfam database, Swiss-Prot database, COG database, eggNOG database and KOG database, respectively. In addition, some randomly selected genes were used to validate the accuracy of the RNAseq data. The results showed that the RNAseq data were equal to qRT-PCR data, proving that the RNAseq data are reliable (Figure S1).

### 2.2. Trichome-Specific Gene Analysis in A. annua

There are several reliable methods for measuring tissue expression specificity, including Tau, Gini, tissue specificity index (TSI), Counts, expression enrichment (EE), z-score, SPM and Preferential Expression Measure (PEM) [19]. Among them, only SPM, z-score, PEM and EE can recognize the specificity of the gene for each tissue. However, z-score is generally applied for features other than tissue specificity, PEM is suggested for ESTs, and SPM’s stability and interpretability are better than EE’s [19,20,21]. Therefore, the SPM formula was applied to the transcriptome data of trichome-expressive genes in *A. annua*. SPM is valued between 0 and 1, corresponding to the housekeeping gene and tissue-specific gene, respectively [22]. Using the expression specificity metric SPM, we found 6646 genes had trichome specificity with SPM > 0.7.

**Figure 1:**
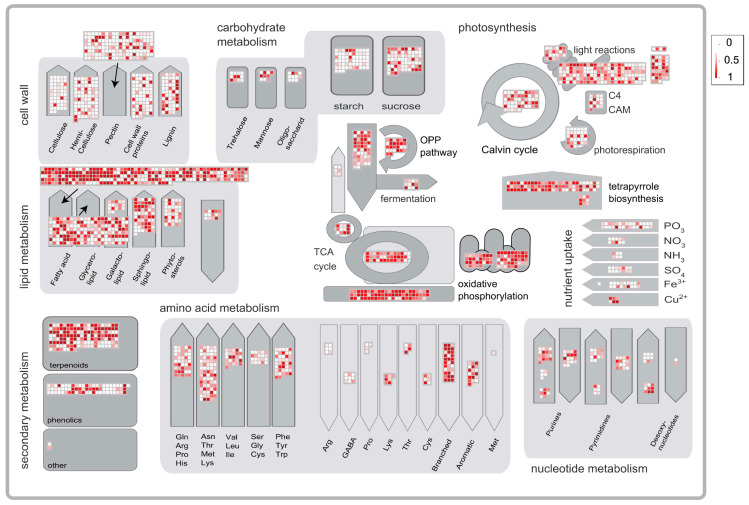
MapMan visualization of metabolism-related gene-specific expression in trichome of *A. annua*. The SPM values of significantly trichome-expressive genes were visualized in MapMan. Each square corresponds to a gene. Red squares indicate genes specifically expressed in the trichome. White squares indicate genes non-specific expressed in the trichome.

The metabolic pathway analysis of tissue-specific tendency genes was performed via Mercator and MapMan. General metabolism analysis demonstrated that genes participating in lipid metabolism, terpenoids, photosynthesis, oxidative phosphorylation, TCA cycle and tetrapyrrole biosynthesis have a highly specific expression in trichomes. In particular, the fatty acid in lipid metabolism contained the largest number of trichome-specific genes, followed by terpenoid metabolism (Figure 1).

### 2.3. Trichome-Specific Gene Involved in Terpenoid Metabolism

**Figure 2:**
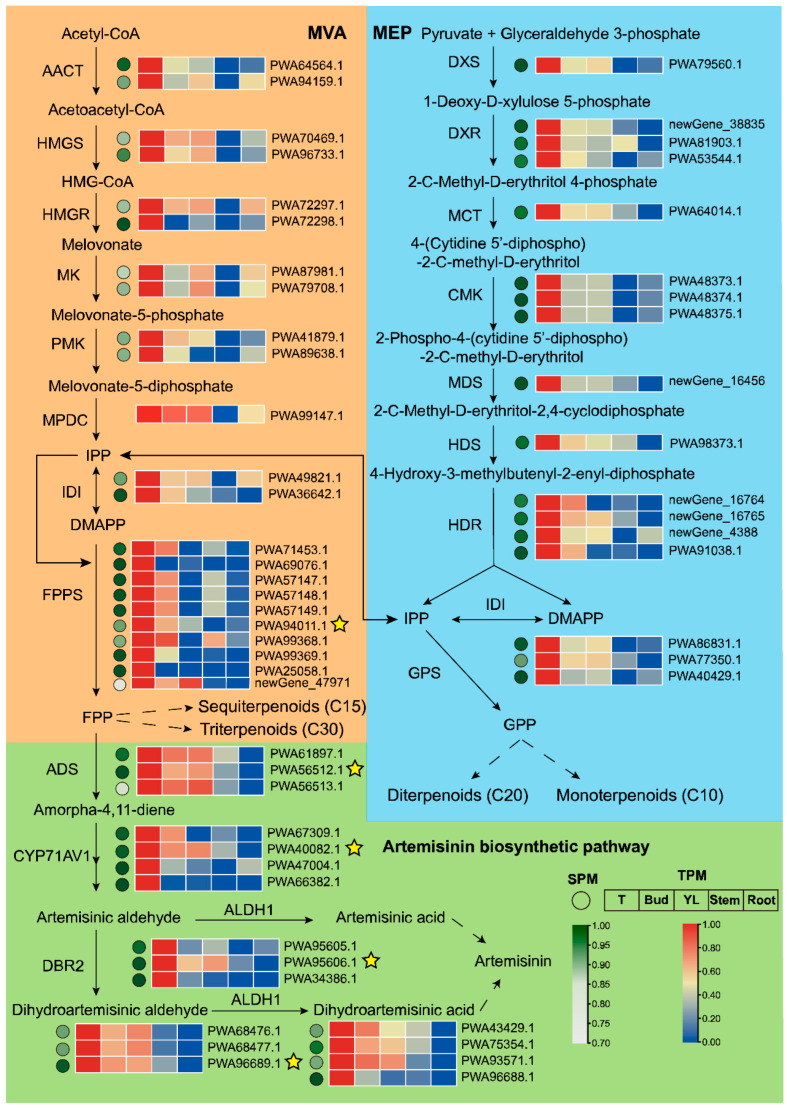
The biosynthesis pathway of trichome-specific terpenoids in *A. annua*. The dashed arrow denotes multiple steps, and the solid arrow is a single step. Red and blue represent high and low expression levels, respectively. Green and white rounds indicate genes significantly specifically and slightly specifically expressed in the trichome, respectively (SPM reflects the degree of trichome-specificity). All function-characterized genes are indicated by yellow stars, which are *PWA94011.1* (*FPPS*), *PWA56512.1* (*ADS*), *PWA40082.1* (*CYP71AV1*), *PWA95606.1* (*DBR2*) and *PWA96689.1* (*ALDH1*) (Shen et al., 2018). Abbreviations: T, trichome; YL, young leaf; AACT, acetoacetyl-coenzyme A thiolase; HMGR, HMG-CoA reductase; HMGS, 3-hydroxy-3-methylglutaryl (HMG)-CoA synthase; PMK, phosphomevalonate kinase; MK, mevalonate kinase; MPDC, mevalonate diphosphate decarboxylase; DMAPP, dimethylallyl diphosphate; IPP, isopentenyl diphosphate; IDI, IPP/DMAPP isomerase; FPP, farnesyl diphosphate; FPPS, farnesyl diphosphate synthase; DXR, 1-deoxy-D-xylulose-5-phosphate reductoisomerase; DXS, 1-deoxyxylulose-5-phosphate synthase; CMK, 4-diphosphocytidyl-2-Cmethyl-D-erythritol kinase; MCT, 2-C-methyl-D-erythritol 4-phosphate cytidylyltransferase; MDS, 2C-methyl-D-erythritol 2,4-cyclodiphosphate synthase; HDR, 4-hydroxy-3-methylbut-2-enyl diphosphate reductase; HDS, 4-hydroxy-3-methylbut-2-enyl diphosphate synthase; GPP, geranyl diphosphate; GPS, geranyl diphosphate synthase; ADS, amorphadiene synthase; DBR2, artemisinic aldehyde reductase; ALDH1, artemisinic aldehyde dehydrogenase.

Based on the annotation of each gene, genes related to terpenoid pathways were collected. Only trichome-specific genes (SPM ≥ 0.7) were selected for expression tendency analysis, shown as a heatmap in Figure 2. Except mevalonate diphosphate decarboxylase (MPDC) (SPM and Tau value for the relevant gene *PWA99147.1* were 0.69 and 0.54), each enzyme in terpenoid-related pathways can be encoded by trichome-specific genes. Most (more than 90%) even had an SPM value greater than 0.90, indicating that genes encoding for terpenoid pathways had a significant trichome specificity. *FPPS* also showed the highest trichome specificity, owing to the largest number of trichome-specific genes and a high SPM value, contributing to C_15_ terpenoid backbone synthesis in the MVA pathway and providing abundant FPP for the production of terpenoids downstream (Figure 2). In addition, many genes in the artemisinin pathway were highly expressed in buds and young leaves, such as *HMGS*, *MPDC*, *ADS* and *ALDH1*, meaning that trichomes in buds and young leaves were the primary tissues for artemisinin biosynthesis. According to our in-house RNAseq data, both terpenoid backbone biosynthetic genes and artemisinin biosynthetic genes (*ADS*, *CYP71AV1*, *DBR2* and *ALDH1*) showed high expression in flowers or young leaves, and low expression in stems (Figure S2). The trichome specificity of the terpenoid pathway was matched with previous studies [15,18,23,24], proving that the data used in our study were credible.

### 2.4. Enrichment Analysis of the Trichome-Specific Expression Genes

**Figure 3:**
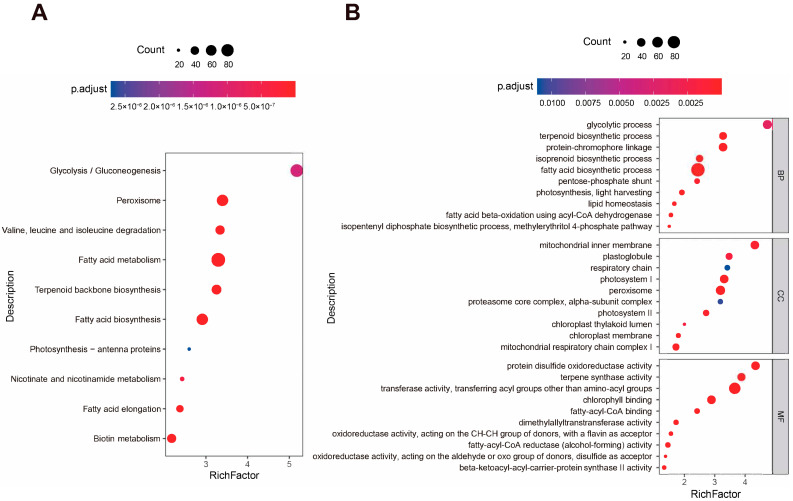
Enrichment analysis of trichome-specific expression genes in *A. annua* trichome. (**A**) KEGG enrichment analysis of genes specifically expressed in trichomes. (**B**) GO enrichment analysis of genes specifically expressed in trichomes. Only the top 10 terms are selected, ordered by the *p*-adjusted value. Terms shown in the figure are ordered by richfactor. Red suggests significantly enriched, and blue represents insignifTableicantly enriched.

To explore the specific biological processes in *A. annua* trichomes, KEGG and GO enrichment analyses were executed. 6646 candidate genes were significantly enriched in 23 KEGG pathways (Table S1) and 71 GO terms (Table S2), including 32 molecular functions, 24 biological processes and 15 cellular components. The most abundant enriched pathway was the glycolysis/gluconeogenesis pathway (rich factor: 5.18), likely due to producing energy for other reactions enriched in trichomes. Similarity, the valine, leucine and isoleucine degradation pathway (rich factor: 3.34) and the nicotinate and nicotinamide metabolism pathway (rich factor: 2.43) were enriched in trichomes. The valine, leucine and isoleucine degradation pathway yields NADH (nicotinamide adenine dinucleotide) and FADH2 (flavine adenine dinucleotide, reduced) for ATP generation, while the nicotinate and nicotinamide metabolism pathway takes part in NAD+ and NADP+ (nicotinamide-adenine dinucleotide phosphate) synthesis. In addition, the enrichment of the photosynthesis pathway (rich factor: 2.60) also allowed the absorption of light energy. Interestingly, as the related pathway of valine, leucine and isoleucine degradation, terpenoid backbone biosynthesis (rich factor: 3.25) was also significantly enriched in trichomes, suggesting the diversity of terpenoids generated in trichomes. In addition, many pathways relating to fatty acids were also abundant, such as the peroxisome pathway (rich factor: 3.40), fatty acid metabolism (rich factor: 3.30), fatty acid biosynthesis (rich factor: 2.91), fatty acid elongation (rich factor: 2.38) and biotin metabolism (rich factor: 2.18), indicating the active biological processes related to fatty acids in trichomes (Figure 3A).

The results of GO enrichment also showed that fatty acid-related processes (fatty acid biosynthetic process and fatty acid beta-oxidation using acyl-CoA dehydrogenase) and terpenoid-related processes (isoprenoid biosynthetic process, terpenoid biosynthetic process, isopentenyl diphosphate biosynthetic process and methylerythritol 4-phosphate pathway) were significantly enriched in the biological process category (Figure 3B).

### 2.5. Weighted Gene Co-Expression Network Analysis

**Figure 4:**
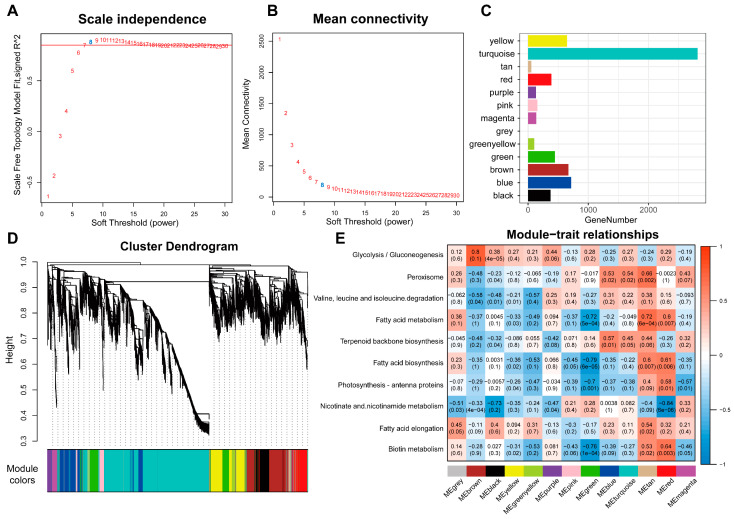
Weighted gene co-expression network analysis of genes specifically expressed in the trichome. (**A**) Scale-free fit index analysis for different soft-threshold powers. The selected soft-threshold power is colored in blue. (**B**) Mean connectivity analysis for different soft-threshold powers. The selected soft-threshold power is colored in blue. (**C**) The number of genes in each module. (**D**) Dendrogram of all expressed genes and corresponding module colors. (**E**) Heatmap of correlation between each module and KEGG pathways.

To obtain a further understanding of genes specifically expressed in the trichome, we created the gene co-expression network to identify gene modules with specific functions through weighted gene co-expression network analysis (WGCNA). Trichome-specific genes (SPM ≥ 0.7) in 19 samples were used in the WGCNA and 13 modules were obtained on the soft-thresholding power of β = 8 (Figure 4). To identify the hub modules related to trichome-specific pathways in *A. annua*, we analyzed the Pearson correlation between enriched KEGG pathways and module eigengenes (ME) using principal component analysis. As shown in Figure 4E, composed of 715 genes, the blue module was identified as significantly correlated with terpenoid backbone biosynthesis (R = 0.57, *p* = 0.01). Artemisinin biosynthesis genes, such as *PWA56512.1* (*ADS*), *PWA40082.1* (*CYP71AV1*), *PWA95606.1* (*DBR2*) and *PWA96689.1* (*ALDH1*), were found in the blue module as well (Table S3). KEGG and GO enrichment showed that key metabolic pathways, including the terpenoid biosynthetic process, flavonoid biosynthetic process and fatty acid pathway, were enriched in the blue module (Figure S3, Tables S4 and S5).

### 2.6. Identification of Trichome-Specific Hub Genes Affecting Artemisinin Synthesis

**Figure 5:**
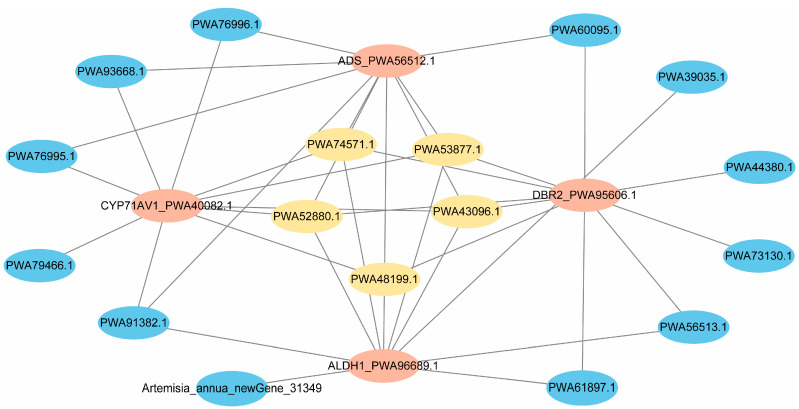
Network for identified hub genes and the correlation of key genes related to artemisinin biosynthesis. The nodes represent the genes, and the edges show the correlation. Hub genes are colored yellow, key genes of artemisinin biosynthesis are colored orange and the other genes are blue.

The correlative interactions in the blue module were selected for further investigation, and the hub interactions with key genes in the artemisinin biosynthetic pathway were determined using the TOM (topological overlap) matrix. The TOM matrix can be used to calculate the extent of similarity by considering both the target genes’ interaction and other interactions including these two target genes, minimizing the influence of noise and false positives [25]. The top 10 interactions of each reported artemisinin biosynthetic gene were selected based on high TOM value (Figure 5). Interestingly, as shown in the network, *PWA43096.1* (*ORA*), *PWA48199.1* (*BAMT*, *Benzoate carboxyl methyltransferase*), *PWA53877.1* (*AATL1*, *Lysine histidine transporter-like 8*), *PWA52880.1* (*putative ulp1 protease family*) and *PWA74571.1* (Tub domain-containing protein) were corelative with all of the five key genes (Figure 5), suggesting the high potential for regulating artemisinin biosynthesis. Among them, through interacting with *TCP14*, a jasmonate-responsive and trichome-specific ethylene-responsive transcription factor, *ORA* regulates the expression of artemisinin biosynthesis genes (*DBR2* and *ALDH1*) [26]. However, the interactions between the other four genes and artemisinin biosynthesis have not yet been reported. The *BAMT* homolog in *Antirrhinum majus* catalyzes the methyl transfer of S-adenosyl-L-methionine (SAM) to the carboxyl group of benzoic acid to form the volatile ester methyl benzoate [27], which belongs to the class of carboxyl methyltransferases. *AATL1* is a transporter, which might facilitate neutral and acidic amino acid transportation across the cellular membrane in higher plants [28]. *Ubiquitin-like protease 1* (*Ulp1*) is a proteolytic enzyme activator in the sumoylation and desumoylation pathways, providing free small ubiquitin-like modifiers (SUMOs) for the conjugation of other proteins. *Ulp1* not only helps to create the mature SUMO peptide by undocking C-terminal amino acid residues of the SUMO precursor, but also functions in helping sumoylated proteins remove the conjugated SUMO peptide [29]. The TUBBY domain is a conserved domain in different species, including mammals (*Mouse* and *Human*) and plants (*Arabidopsis*, *Oryza sativa* and *Zea mays*) [30]. Although reports about tub domain-containing protein are rarely found in plants, experimental data show that the TUBBY protein is a bipartite transcription regulator in mammals [31]. Its homological *PLSCRs* (phospholipid scramblases) family in *Arabidopsis* are Ca^2+^-activated membrane tethered transcription factors [32]. In addition, plant *TLP* (*TUBBY*-like proteins) members have a conserved F-box-containing domain, followed by specific sequences that serve as protein–protein interacting domains in order to recruit specific proteins for ubiquitin-mediated proteolysis [33].

In addition, *PWA91382.1* (*PDR1*, ABC-type transporter family protein) also attracted our attention, which had high connections with *ADS*, *CYP71AV1* and *ALDH1* (Figure 5). In previous studies, we found *PDR1* shared the same expression profiling with artemisinin biosynthetic genes [34]. Additionally, two homolog *ADS* genes have been found in this network: *PWA61897.1* (identities = 90.71%, E-value = 0.00) and *PWA56513.1* (identities = 95.98%, E-value = 2 × 10^−137^).

### 2.7. Quantitative Real Time (qRT)-PCR Validation of Candidate Genes

**Figure 6:**
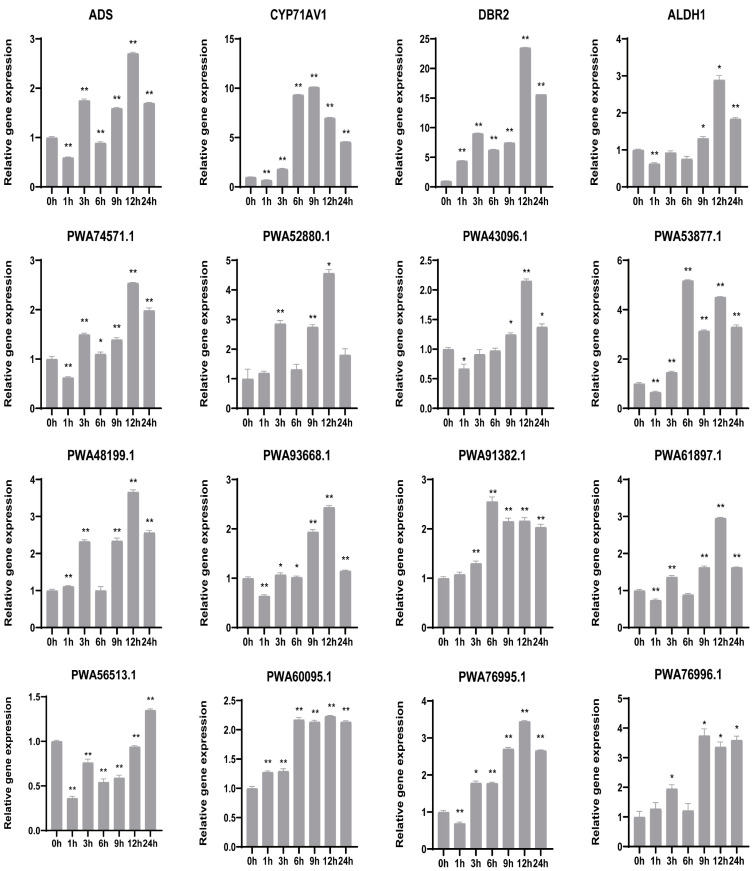
The relative expression levels of artemisinin biosynthetic genes and candidate hub genes in *A. annua* are treated by 100 μM MeJA over 24 h. Averaged from three replicates, the data are further analyzed using *t*-test for significant differences between treatments and control. The error bar means ± SD (n = 3); * and ** indicate that values are significantly different at *p*  <  0.05 and *p*  <  0.01 levels, respectively.

To analyze the responses of the candidate genes to MeJA treatment by qRT-PCR, as shown in Figure 6, the expression levels of artemisinin biosynthetic genes (*ADS*, *CYP71AV1*, *DBR*2, *ALDH1*) and the candidate genes were induced by MeJA. Results showed that five candidate genes (*PWA74571.1*, *PWA48199.1*, *PWA93668.1*, *PWA76995.1* and *PWA61897.1*) and *ADS* shared similar expression profiling during MeJA treatment, which was high at 3 h and 12 h MeJA treatment. Meanwhile, two candidate genes (*PWA60095.1* and *PWA91382.1*) showed high expression from 6 to 24 h, and *PWA76996.1* showed high expression from 9 to 24 h. *ALDH1*, *DBR2* and most candidate genes (such as *PWA43096.1* and *PWA52880.1*) showed the highest expression at 12 h, while *PWA53877.1*, *CYP71AV1* and *PWA56513.1* showed high expression at 6 h, 9 h and 24 h, respectively. After MeJA treatment, the expression of candidate genes was upregulated, indicating that the candidate genes positively respond to the JA signaling to boost artemisinin biosynthesis in *A. annua*.

In brief, hub genes were obtained by correlation network analysis for the key artemisinin biosynthetic genes. In addition to the known *ORA* and *PDR1*, the finding of hub genes *BAMT*, *AATL1*, *UIp1* and Tub domain-containing protein implies the transfer of carboxyl and methyl, the transportation of amino acids and that SUMO’s reactions may involve artemisinin biosynthesis.

## 3. Discussion

Trichomes are important plant tissues for hosting a wide range of secondary metabolic processes; however, their molecular basis is not well understood, especially in *A. annua*. Studying trichome-specific transcripts will contribute to the finding of candidate genes to improve useful trichome-specific secondary chemicals. The innovation in our study was the combined use of high-throughput sequencing data, a tissue specificity algorithm and co-expression network analysis. To better understand secondary metabolic processes at the molecular level, trichome-specific genes were filtered based on the comparison of trichomes with buds, young leaves, stems and roots using RNAseq data and an SPM algorithm. We found that metabolism relevant to energy, terpenoids and fatty acids was enriched in the trichome-specific gene set. Further WGCNA analysis showed that artemisinin-related genes hold a specific expression pattern that clusters in the blue module. The candidate genes interacting with the key function-characterized artemisinin-related genes might be involved in the transfer of carboxyl and methyl, amino acid transportation and SUMO reactions, which are regulated by jasmonic acid (JA) treatment. In addition to providing rich information on trichome metabolic processes, our analysis also sheds light on hub genes that may be of interest to further research.

For trichomes to produce secondary metabolites, primary carbon flux and energy must be sufficient. In the previous study of *Artemisia annua* glandular trichome ESTs, GO terms related to ATP reaction were highly enriched in the *A. annua* unigenes and all trichome ESTs databases [15]. Identical to this report, ATP generation-related pathways were significantly enriched in trichomes in our study, such as TCA cycle, oxidative phosphorylation, glycolysis/gluconeogenesis and photosynthesis. It is worth noting that photosynthesis metabolism cooperates with the JA response pathway and also can activate the promoter of *ADS*, *CYP71AV1*, *DBR2* and *ALDH1* through MYB transcription factors to regulate artemisinin biosynthesis [35]. Trichome-specific transcription factors can regulate artemisinin biosynthesis by mediating light and JA signaling; these include *AaWRKY9* and *AaMYB108* [36,37]. The mechanism of JA and light signal crosstalk in regulating artemisinin accumulation is still unknown [38]. Recent studies showed that the integration of JA and light signaling not only regulate artemisinin biosynthesis but also mediate trichome initiation by enhancing the transcription activation of *AaHD1* on downstream *AaGSW2* [12]. This research also found that the molecular function of transport was the highest enriched GO term in the *A. annua* glandular trichome, with many ABC transporter homologous contigs [15]. Similar to this research, transporters *AATL1* and *PDR1* were found in the analysis of trichome-specific hub genes to be correlated with artemisinin biosynthesis.

Fatty acids and lipids are major and necessary elements of all plant cells; they not only generate energy for abundant metabolic processes but also serve as signal transduction mediators. Genes that took part in lipid biosynthesis, particularly those relevant to the biosynthesis of polyunsaturated fatty acids, showed higher expression in glandular trichomes in many plants, such as *Nicotiana tabacum* [39], *Mentha spicata* [40] and *tomato* [41]. In *A. annua*, compared with the transcriptome of filamentous and glandular trichomes, some lipid biosynthesis-related contigs hold a higher expression in glandular trichomes, covering most of the major steps of fatty acid biosynthesis [17]. Our research also found that many fatty acid–related genes showed trichome specificity through the SPM algorithm and WGCNA. Some clustered in the blue module, which had a close relationship with terpenoid biosynthesis, indicating that fatty acid biosynthesis has the potential to influence the pathways of terpenoids. In addition, similar to reports in other plants (*tomato* [42] and *tobacco* [43]), *lipid transport protein* (*LTP*) genes show high trichome-specific expression (Table S3) and function in lipid intermembrane transfer facilitation, cutin wax formation, plant signaling, germination and lipid metabolism in plants [44]. More importantly, *AaLTP1*, *AaLTP3* and *AaLTP4* might relate to the secretion of sesquiterpene lactone from glandular trichomes [34,45]. In our WGCNA results, most *LTP* genes were also enriched in terpenoid relevant modules. Thus, further research to investigate the mechanism of LTP in terpenoid bioprocesses is needed.

Terpenoid biosynthesis in *A. annua* has been reported to have specifically occurred in glandular trichomes. Thus, researchers have aimed to determine new candidate genes for artemisinin biosynthesis by comparing the gene expression between trichomes and other tissues, or between different trichomes [15,17]. Based on the published genomes and specialized arithmetic for tissue specificity, we analyzed the expression of trichome-specific genes in the terpenoid backbone pathway (MEP and MVA) and artemisinin pathway, including the genes that have been function-characterized. As a result, we proved the trichome specificity of terpenoids and obtained a high-quality trichome-specific gene set. Interestingly, both our study and Soetaert et al. found genes related to flavonoid biosynthesis had a close relationship with terpenoid biosynthesis [17]. They identified two dioxygenases flavanone 3-hydroxylase (*F3H*) contigs as potential candidate genes for improving artemisinin biosynthesis [17]. We not only found *F3H* genes but also some genes encoding for isoflavone reductase homology, flavonol synthase, flavonone isomerase, 2-alkenal reductase and chalcone synthase in the blue module through WGCNA analysis, which were significantly related to terpenoid biosynthesis (Table S3). The plant’s acclimation to changing environmental conditions is often aided by flavonoids and terpenoids. Studies in tomatoes have shown that flavonoid biosynthesis can regulate terpenoid biosynthesis in glandular trichomes [46]. Evidence shows that metabolic activity of flavonoid and terpenoid pathways is coordinated by MYB family transcription factors. We also found *PtMYB14* homological gene *PWA70731.1* (identities = 50.62%, E-value = 2 × 10^−55^) in the blue module, which possibly acts as the bond between terpenoid and flavonoid biosynthesis [47]. At the same time, *PWA70731.1* was also characterized as the homological gene of *AaMYB1* (identities = 62.93%, E-value = 2 × 10^−55^); it functions in trichome development and terpenoid metabolism [48]. Interestingly, the relationship between lipid metabolism and flavonoid production in secondary metabolism has been revealed as acetyl-CoA carboxylase (*ACC*), involved in the biosynthesis of flavonoid and long-chain fatty acid [49], implying that lipid also has the potential to affect terpenoid biosynthesis. Although some potential connections between terpenoid and fatty acid processes in trichomes were revealed in our study, we were unsuccessful in further proving the associations of trichome-specific genes involved in different metabolisms due to insufficient sample size; this is an important issue for future research.

Moreover, post-translational modifications (PTMs) may play a vital role in trichome-specific metabolic processes, including phosphorylation, ubiquitination and methylation [50]. As shown in the MapMan result, the phosphorylation is enriched in the trichome-specific gene set, which is controlled by the antagonism of protein kinases and phosphatases and regulates key cellular processes, participating in intercellular communication and coordination. The introduction of a phosphoryl group affects protein–protein interactions and/or enzymatic activity through changes in conformation [51]. Hub genes related to SUMO reactions indicate that ubiquitination is significant in artemisinin biosynthesis, as reversible modifications that can rapidly activate or invalidate the target’s molecular function [52]. SUMO modification (SUMOylation) can influence protein stability, protein function, protein targeting, enzyme activity, protein–protein interactions and cellular localization. Recent research has shown that metabolism can be affected by SUMOylation. In mammals, as a major controller of key genes for fatty acid, phospholipid and triacylglyceride biosynthesis, as well as the production of acetyl-CoA cofactors and NADPH, transcription factor *SREBP-1a* (sterol regulatory element binding proteins 1a) is regulated by two PTMs, phosphorylation and SUMOylation [53]. Therefore, it is possible that SUMO can affect artemisinin biosynthesis by regulating the activity of transcription factors or other related proteins in *A. annua.* The methyltransferase gene was also found as a hub gene related to artemisinin biosynthesis, which dynamically controls the methylation condition of target proteins with demethylases. One of the epigenetic modifications occurs in histones when the methyl group bonds to the arginine and lysine residues, which affects the chromatin structure and non-histone recruitment [50]. Therefore, DNA methylation is significant for genome stability and gene regulation, contributing to the regulation of gene expression, chromosome interactions, transposon silencing, and even stress responses and plant development [54]. Presumably, methyltransferase may inhibit the combination of some transcription repressors or enhance the interactions of some transcription activators related to artemisinin biosynthesis by promoting DNA methylation. However, publications regarding PTMs in plant metabolism are rare; thus, it will be interesting to further investigate the role of PTMs in artemisinin biosynthesis in trichomes.

## 4. Materials and Methods

### 4.1. Resources Used for Data Analysis

Reference genome (accession: GCA_003112345.1) and RNA sequencing data of trichomes, flowers, buds, young-leaves, stems, roots and seeds (accession: SRR6472949, SRR6472948, SRR6472946, SRR6472941, SRR6472943, SRR6472944 and SRR6472947) were obtained from NCBI.

### 4.2. Plant Material Collection and RNAseq

*Artemisia annua*, named “Huhao 1”, was used in this study to verify the transcriptome from the previous study and identify novel genes in the trichome. It was cultivated in the climate chamber of the South China Agricultural University (23° N, 113° E). Flowers, young leaves, old petioles and stems were collected from 6-week-old mature plants and frozen in liquid nitrogen. Samples of each tissue were ground into powder and divided into three replications (300 mg). Afterward, the samples were stored at −80 °C for further use.

To measure RNA concentration and purity, NanoDrop 2000 (Thermo Fisher Scientific, Wilmington, DE, USA) was used. With the RNA Nano 6000 Assay Kit of the Agilent Bioanalyzer 2100 system (Agilent Technologies, Santa Clara, CA, USA), RNA integrity was evaluated. Following the manufacturer’s recommendations, sequencing libraries were constructed using the NEBNext UltraTM RNA Library Prep Kit for Illumina (NEB, Ipswich, MA, USA). In brief, using poly-T oligo-attached magnetic beads and divalent cations under elevated temperature in NEBNext First Strand Synthesis Reaction Buffer (5X) to purify mRNA from total RNA and randomly broken RNA. Then, cDNA was reverse transcribed using random hexamer primer, M-MuLV Reverse Transcriptase, DNA Polymerase I and RNase H. Using the AMPure XP system (Beckman Coulter, Indianapolis, IN, USA), the library fragments were purified. Then, 3 μL USER Enzyme (NEB, Ipswich, MA, USA) was used with size-selected, adaptor-ligated cDNA at 37 °C for 15 min followed by 5 min at 95 °C before PCR. Then, PCR was applied using Phusion High-Fidelity DNA polymerase, Universal PCR primers and Index (X) Primer. On the Agilent Bioanalyzer 2100 system, PCR products were purified (AMPure XP system, Beckman Coulter, IN, USA), and library quality was evaluated.

Using TruSeq PE Cluster Kit v4-cBot-HS (Illumina, San Diego, CA, USA), the clustering of the index-coded samples was executed on the cBot Cluster Generation System. Then, the library preparations were sequenced on the Illumina platform, and paired-end reads were produced.

### 4.3. Quantitative Real Time (qRT)-PCR

Fifteen-day-old *Artemisia annua* seedlings were treated with 100 mM MeJA and collected at 0, 1, 3, 6, 9, 12 and 24 h for qRT-PCR. Around 100 mg of the frozen ground leaves were used for total RNA extraction and removal of genomic DNA contamination using RNAprep Pure Plant Kit (TIANGEN, Beijing, China). The collected RNA from *A. annua* samples were reverse transcribed into complementary DNA (cDNA) using a PrimeScript RT reagent Kit (TaKaRa, Dalian, China). All of the primers were designed on the website Primer3Plus (https://primer3plus.com/ (accessed on 30 December 2022)) (Table S6), and the specificity of the primer was tested by Primer-BLAST (http://www.ncbi.nlm.nih.gov/tools/primer-blast/ (accessed on 30 December 2022)). The qRT-PCR was analyzed using an AnalytikJena AG qTOWER 3G (AnalytikJena, Jena, Germany) (Reference gene: 18S rRNA, three replicates for each sample). The 2^−ΔΔCt^ method was used to calculate the relative expression levels of the test genes.

### 4.4. Quality Control and Alignment of Sequencing Datasets

Raw data of RNAseq was transformed into clean reads through in-house perl scripts and then mapped to the reference genome sequence. HISAT 2.2.0 software (2020) was used to map the reference genome [55], and mapped reads were assembled using StringTie [56]. Quantification of gene expression levels was estimated by fragments per kilobase of transcript per million fragments mapped (FPKM). In addition, raw data of the NCBI datasets were downloaded as fastq files by Aspera. To confirm the quality of the sequence reads, the quality control tool FastQC v0.11.9 was used. The adapters and low-quality reads were removed by using Trimmommatic 0.39. The expression levels of transcripts were quantified using Salmon, based on our own gene set of the transcriptome [57]. Transformation of the transcript per million (TPM) and the FPKM was accomplished via the Expression Calculate program of TBtools [58].

### 4.5. Gene Annotation and Enrichment Analysis

Using BLAST, the gene function was annotated against the following databases: Swiss-Prot (a manually annotated and reviewed protein sequence database); Nr (NCBI non-redundant protein sequences); GO (Gene Ontology Resource); KO (KEGG ortholog database). KEGG orthology of new genes was identified using KOBAS2.0 [59]. Subsequently, using an R package, clusterProfiler v4.4.4, the genes highly expressed in trichomes were subjected to KEGG and GO enrichment analysis [60].

### 4.6. Calculation of Trichome Specificity Index

In our study, the SPM index was used to perform the trichome specificity of each gene, calculated by tspex web application (https://tspex.lge.ibi.unicamp.br/ (accessed on 15 November 2022)) [61]. The SPM index measures how specifically or broadly a gene is expressed, with a value of 1 suggesting expression that is specific to just one tissue, and a value of 0 indicating equal expression throughout all tissues [22]. The MapMan program was used to visualize the trichome-specificity of genes in various metabolisms [62]. Before using this tool, functional bins were assigned using Mercator4 v5.0 (default options) [63].

### 4.7. Expression Pattern Analysis

The function of Heatmap in TBtools was used to visualize the trend of expression in the trichome, bud, young leaf, stem and root using the TPM value of each gene [58]. In addition, to verify the accuracy of previous expression data, bar graphs showing the TPM value of each gene were displayed by the ggplot2 v3.3.6 R-package [64].

### 4.8. Weighted Gene Co-Expression Network Analysis

To identify trichome-specific modules and their members, WGCNA was analyzed by the R WGCNA package (v1.71) [25]. A suitable soft-thresholding power (β = 8) was selected through the pickSoftThreshold function in the WGCNA package. Using the Dynamic Hybrid Tree Cut algorithm, genes were classified into different modules based on expression patterns and represented by different colors. In addition, to identify gene modules with substantial associations for further analysis, the relationships between the WGCNA modules and the top 10 terms enriched in KEGG analysis were determined.

### 4.9. Identification of Hub Genes

To identify the hub genes, we transformed the expression matrix into a topological matrix and used the average linkage hierarchical cluster method to cluster genes based on TOM. According to the high TOM value, the top 10 interactions of each reported artemisinin biosynthetic genes (*PWA56512.1*, *PWA40082.1*, *PWA95606.1* and *PWA96689.1*) were selected and visualized in Cytoscape [65].

## 5. Conclusions

Trichome-specific expressed genes in *A. annua* were explored using the transcriptome data with the SPM algorithm. Such transcripts were enriched in fatty acid–related pathways and the terpenoid biosynthetic process and photosynthesis pathway. It is still not known whether the crosstalk among these metabolic pathways affects artemisinin accumulation. Further WGCNA analysis identified the module that correlated with terpenoid backbone biosynthesis and characterized the putative hub genes, providing putative candidate genes in regulating artemisinin biosynthesis and accumulation. In addition, some of hub genes might be involved in the transfer of carboxyl and methyl, amino acid transportation and SUMO reactions, making the effect of post-translational modifications apparent. The investigation of how post-translational modifications contribute to artemisinin biosynthesis is a topic for future research.

## Data Availability

The data that support the findings of this study are openly available in NCBI (BioProject ID: PRJNA916469).

## Supplementary Materials

The following supporting information can be downloaded at: https://www.mdpi.com/article/10.3390/ijms24108473/s1.
