# Supplementary material for: Trichome-Specific Analysis and Weighted Gene Co-Expression Correlation Network Analysis (WGCNA) Reveal Potential Regulation Mechanism of Artemisinin Biosynthesis in Artemisia annua
Source: Int J Mol Sci. 2023 May 9;24(10):8473. doi: 10.3390/ijms24108473 (PMC10217836; doi:10.3390/ijms24108473)
Supplement: Supplementary file 1 [file ijms-24-08473-s001.zip › ijms-2368722-supplementary/Supp fig.pdf]

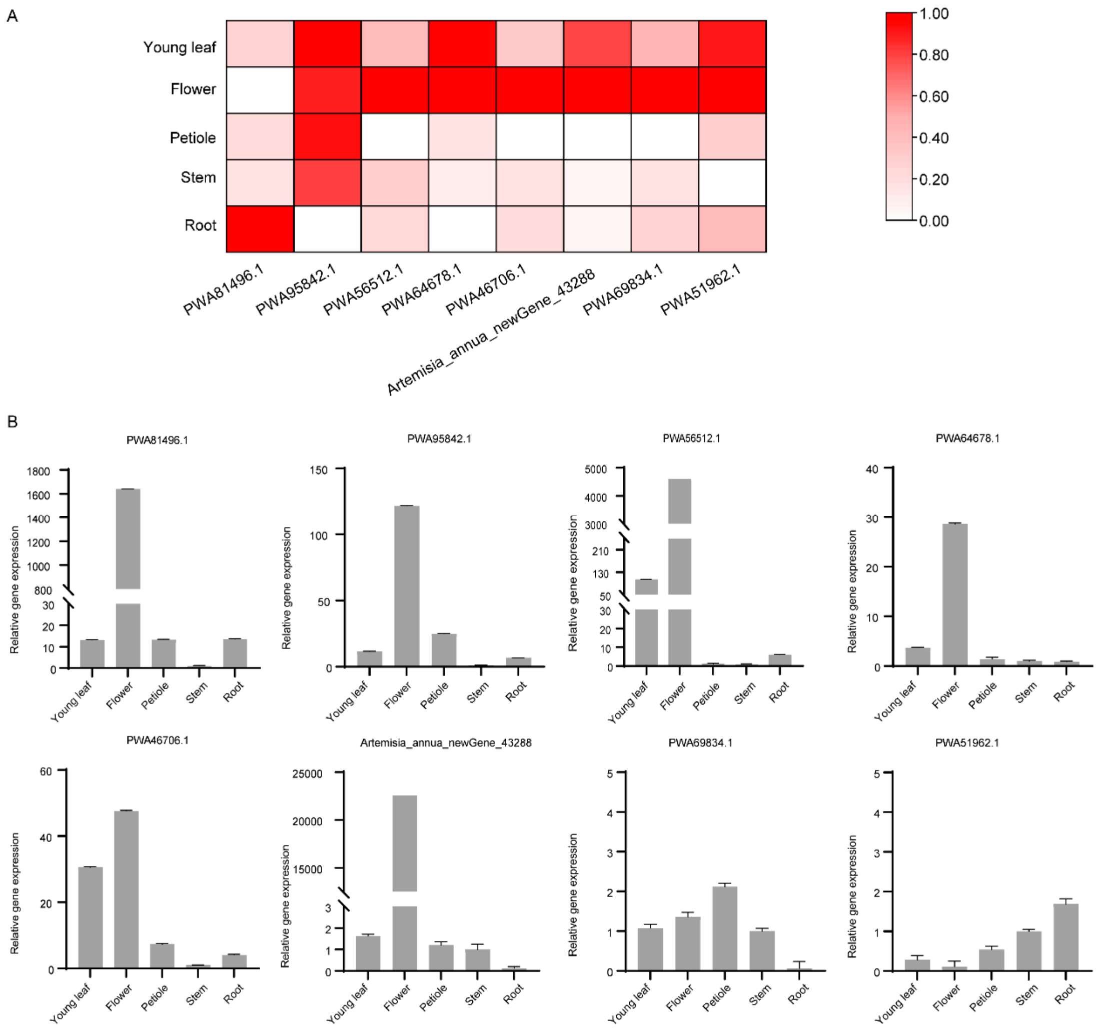

Figure S1. Validation of RNA-seq data using the random selected genes. A, heatmap of the random selected genes in RNAseq data; B, qRT-PCR analysis of the random selected genes in different tissues of *A. annua*. Relative expression levels were calculated using the $2^{(-\triangle\triangle Ct)}$ method, Error bars represent the standard deviations from three biological replicates.

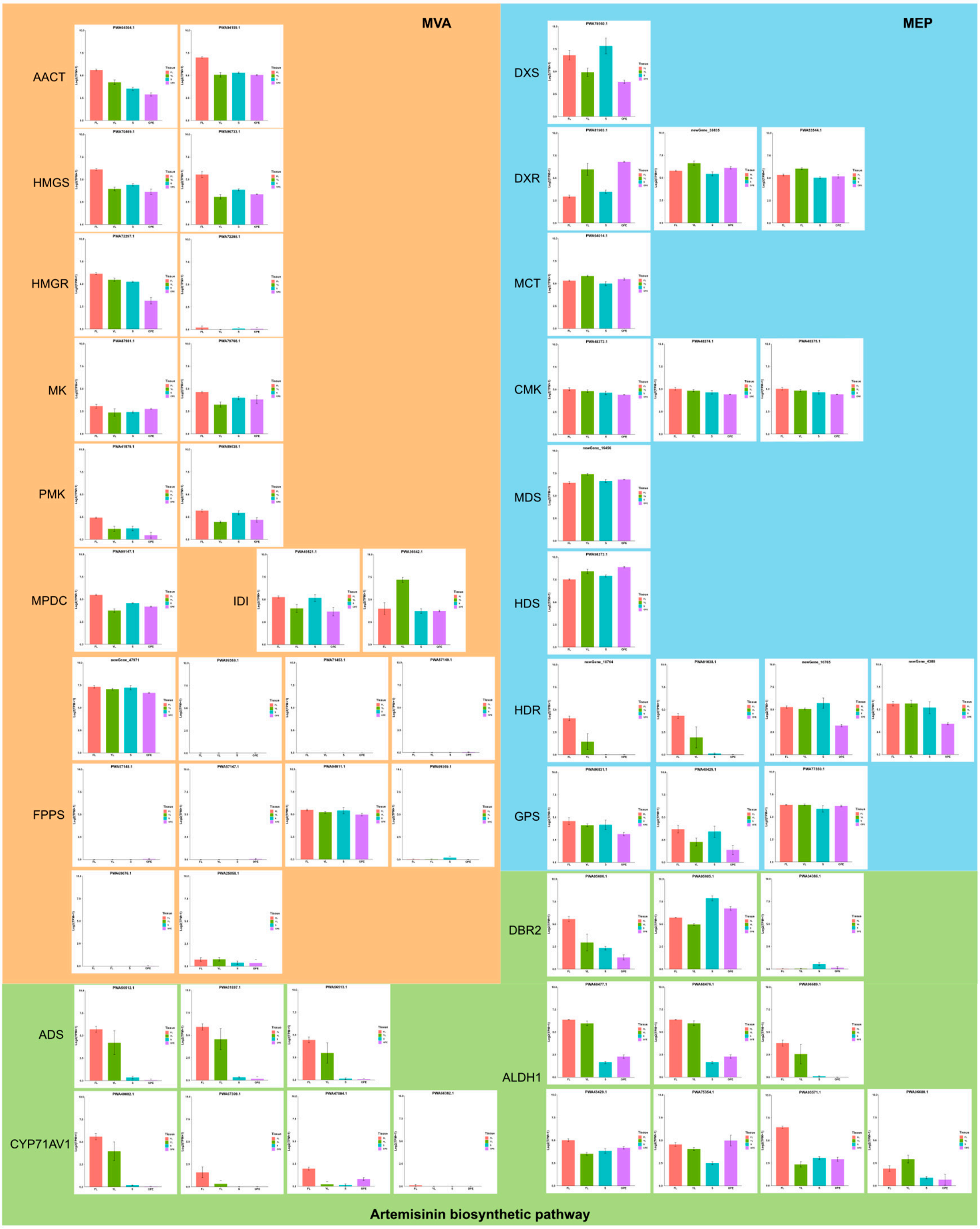

Figure S2. In-house RNA-seq data of the biosynthesis pathway of trichome-specific terpenoids in *A. annua*. Red, green, blue and purple represent the gene's expression in flowers, young leave, stem and old petioles, respectively.

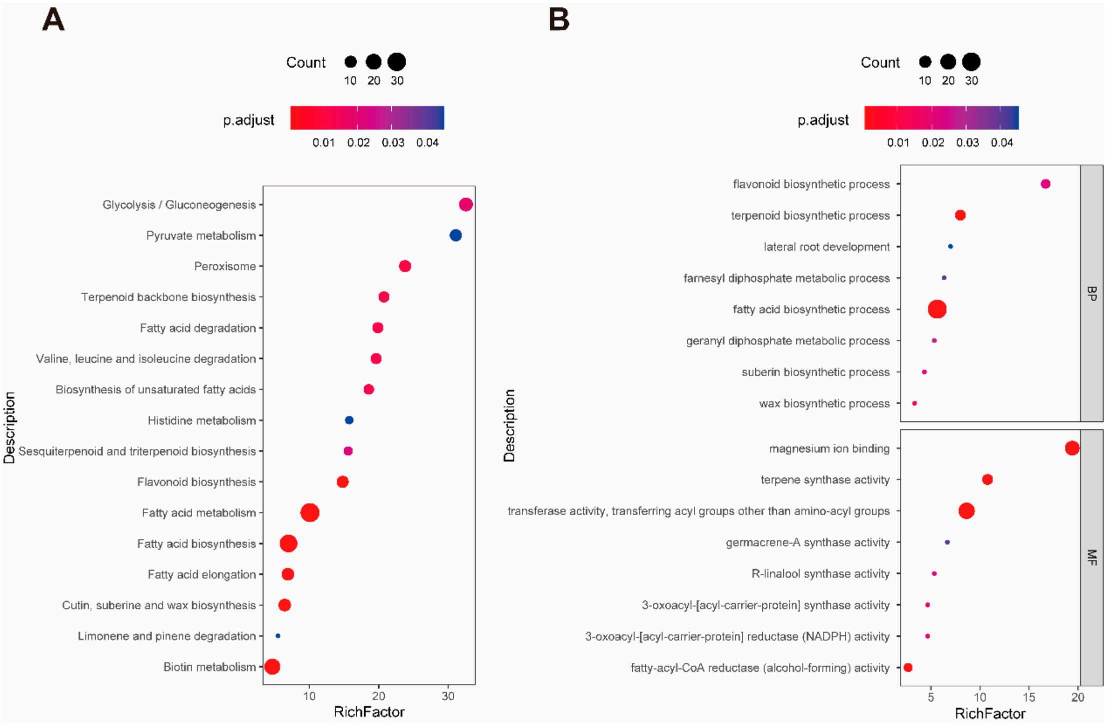

Figure S3. Enrichment analysis of genes in blue module of WGCNA analysis. (A) KEGG enrichment analysis of genes in blue module. (B) GO enrichment analysis of genes in blue module. Only top 10 terms are selected, ordered by *p*.adjust value. Terms shows in figure are ordered by richfactor. Red suggests significantly enriched, and blue represent insignificantly enriched. None of cellular components term significantly enriched in blue module.
